# Non-covalent immunoproteasome inhibitors induce cell cycle arrest in multiple myeloma MM.1R cells

**DOI:** 10.1080/14756366.2019.1594802

**Published:** 2019-07-16

**Authors:** Roberta Ettari, Giovanni Pallio, Gabriele Pizzino, Natasha Irrera, Maria Zappalà, Santina Maiorana, Carla Di Chio, Domenica Altavilla, Francesco Squadrito, Alessandra Bitto

**Affiliations:** a Department of Chemical, Biological, Pharmaceutical and Environmental Sciences, University of Messina, Messina, Italy;; b Department of Clinical and Experimental Medicine, University of Messina, Messina, Italy;; c Department of Biomedical Sciences, Dentistry, and Morphofunctional Sciences, University of Messina, Messina, Italy

**Keywords:** Immunoproteasome, multiple myeloma, cyclins

## Abstract

Proteasome inhibition is a promising strategy for the treatment of multiple myeloma; unfortunately, this disease is often associated with an increasing chemoresistance. One novel approach may be to target the immunoproteasome, a proteasomal isoform mainly present in cells of hematopoietic origin. We investigated the activity of a panel of amides against immunoproteasome core particles as potential agents for the treatment of multiple myeloma (MM). Amide **6** showed an ideal profile since it was able to inhibit both the chymotrypsin-like activities of the immunoproteasome with *K*_i_ values of 4.90 µM and 4.39 µM for *β*1i and *β*5i, respectively, coupled with an EC_50 _=17.8 µM against MM.1R cells. Compound **6** inhibited also ubiquitinated protein degradation and was able to act on different phases of MM cell cycle reducing the levels of cyclin A/CDK1, cyclin B/CDK1 and cyclin D/CDK4/6 complexes, which turns in cell cycle arrest.

## Introduction

The 20S proteasome is the major non-lysosomal proteolytic system in eukaryotic cells, and it plays a key role in the degradation of most cellular proteins. Proteasome is characterised by a barrel-like structure, composed of four stacked rings, each of which contains seven subunits. The two outer rings contain α subunits (α1–α7), while the two inner rings β subunits (β1–β7). The catalytic sites are associated with the β1c, β2c and β5c subunits, which are responsible for the caspase-like (C-L), trypsin-like (T-L) and chymotrypsin-like (ChT-L) activities, respectively^1^.

Vertebrates possess, in addition to the constitutive proteasome, a specialised form of proteasome, named immunoproteasome (i20S), predominantly expressed in monocytes and lymphocytes, and responsible for the generation of major histocompatibility complex (MHC) class I ligands^2^. Under the stimulation of IFN-γ and TNF-α, the constitutive core particles are replaced by the newly formed immunosubunits: β5i (LMP7), β1i (LMP2) and β2i (MECL-1)^3^. While β5i and β2i maintain the same type of activities than β5c and β2c subunits, on the contrary, β1i mainly performs a ChT-L activity, thus cleaving peptides after hydrophobic amino acids.

i20S is the major form of the proteasome expressed in cells of hematopoietic origin, in particular in multiple myeloma (MM) cells^4^; however, Parlati et al. demonstrated that a specific inhibition of either β5i or β5c alone is insufficient to produce an antitumor response, differently, a selective inhibition of both β5i and β5c is fundamental to promote an antitumor effect in MM, non-Hodgkin lymphoma, and leukaemia cells with a reduction of the toxicity in non-tumour cells^5^.

Our research group has been involved in the last years in the development of novel peptidomimetics as inhibitors of the ChT-L of activity of constitutive proteasome^6–11^, in particular we focused our efforts on non-covalent inhibitors, which might be a promising alternative to use in therapy, because of the lack of all side-effects which may be related to irreversible inhibition.

**Figure 1.**
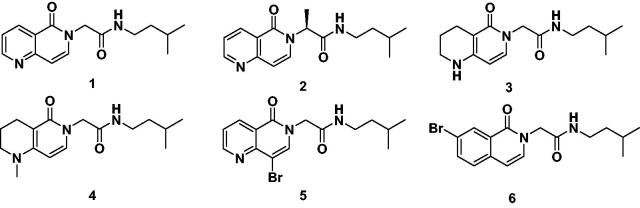
Structure of peptidomimetic amides **1**–**6**.

In this context, we decided to investigate the activity against immunoproteasome core particles of a panel of amides (i.e. **1–6**), previously synthesised by our research group^8^. The selected amides were characterised by the presence of a constrained motif represented by a bicyclic 1,6-naphthyridin-5(6*H*)-one or isoquinolin-1(2*H*)-one scaffold at the P3 site; a glycine or an alanine residue was introduced at the P2 site, while the isopentylamine, projecting into the S1 pocket, was kept unchanged as P1 moiety (Figure 1). Some of the peptidomimetic amides were proven to be active against the ChT-L activity of constitutive proteasome^8^: more in detail, compound **3** showed a *K*_i_ value of 10.5 µM against β5c subunit, while amides **5** and **6** inhibited the β5c catalytic activity with *K*_i_ values of 0.56 and 2.34 µM, respectively ^8–9^.

In this work, we now evaluated the profile of amides **1**–**6** against the immunoproteasome core-particles. The effects of active amides on immunoproteasome subunits were also evaluated in a cellular context, using the dexamethasone-resistant (MM.1R) human multiple myeloma cells. Efficacy of these compounds in inhibiting ubiquitinated protein degradation was also evaluated.

Considering that cell division is the interplay of several cell cycle regulatory proteins, we analysed the ability of active inhibitors to modulate the expression of cyclins, cyclin-dependent kinases (CDKs) and transcription factors, involved in cell cycle check points.

## Materials and method

### *In vitro* 20S immunoproteasome inhibition assays

Human 20S immunoproteasome, isolated from human spleen, was purchased from Enzo Life Science Supplier. The three distinct proteolytic activities of the 20S immunoproteasome were measured by monitoring the hydrolysis of the peptidyl 7-amino-4-methyl coumarin substrates Suc-Leu-Leu-Val-Tyr-AMC (Bachem), Boc-Leu-Arg-Arg-AMC (Bachem) and Ac-Pro-Ala-Leu-AMC (Biomol GmbH) for β5i, β2i and β1i activities of immunoproteasome, respectively. The preliminary screening for the inhibition of the three proteolytic activities of the 20S immunoproteasome was performed at 100 µM inhibitor concentrations using an equivalent amount of DMSO as a negative control, and MG-132 as positive control. Compounds showing at least 70% inhibition at 100 µM were subjected to detailed assays. The dissociation constants *K*_i_ of the enzyme–inhibitor complex were obtained from progress curves (10 min) at various concentrations of inhibitor by fitting the progress curves to a 2 parameter IC_50_ equation, and correction to zero substrate concentration from *K*_i_=IC_50_/(1 + [S] *K*_m_^−1^). Inhibitor solutions were prepared from stocks in DMSO. Each independent assay was performed twice in duplicate in 96-well-plates in a total volume of 200 µL. Fluorescence of the product AMC of the substrate hydrolyses was measured using an Infinite 200 PRO microplate reader (Tecan, Männedorf, Switzerland) at 30 °C with a 380 nm excitation filter and a 460 nm emission filter.

### Assaying the chymotrypsin-like activities (β5i and β1i) of the 20S immunoproteasome

Human 20S immunoproteasome was incubated at 30 °C at a final concentration of 0.004 mg/mL with test compound present at variable concentrations. The reaction buffer consisted of 50 mM Tris HCl, pH 7.4, 10 mM NaCl, 25 mM KCl, 1 mM MgCl_2_, 0.03% SDS. Product release from substrate hydrolysis (50 µM) was monitored continuously over a period of 10 min.

### Assaying the trypsin-like activity (β2i) of the 20S immunoproteasome

Human 20S immunoproteasome was incubated at 30 °C at a final concentration of 0.004 mg/mL with test compound present at variable concentrations. The reaction buffer consisted of 50 mM Tris HCl, pH 7.4, 50 mM NaCl, 0.5 mM EDTA III, 0.03% SDS. Product release from substrate hydrolysis (50 µM) was monitored continuously over a period of 10 min.

### Assessment of cytotoxicity by MTT assay

To test the efficacy of **5** and **6** a cell viability assay was performed. Human multiple myeloma cells (MM.1R; ATCC® CRL-2975™) resistant to dexamethasone were purchased by LGC Standards (Milan, Italy) and cultured under standard conditions (37 °C, 5% CO_2_) with standard RPMI 1640 medium added with L-glutamine, 10% foetal bovine serum, and 1% antibiotic mixture (penicillin/streptomycin) in sterile flasks. Cells were grown for a week before the assay. Compounds **5**–**6** were tested at different doses (1–10-20–40-80–100 µM) in a 96 well plate for 24 h to determine the cytotoxic effect. The tetrazolium dye MTT 3–(4,5-dimethylthiazol-2-yl)-2,5-diphenyltetrazolium bromide (Sigma Aldrich, Milan, Italy) dissolved in sterile-filtered PBS at the dose of 5 mg/ml was added to the plate (20 µl/well) 5 h before the end of the 24 h and the plates were returned into the incubator. At the end of the 5 h, the medium was removed and the insoluble formazan crystals were dissolved with 200 µl/well of dimethyl sulphoxide (DMSO), and the absorbance was read at 540 and 620 nm. The difference between the two absorbance values was used to calculate mean for each replicate and further assess cytotoxicity. All doses were tested in duplicate, negative controls (cells without the tested compounds) were in quadruplicate in each plate, and all the experiment was repeated 5 times. Results were expressed as % of vitality and reported as means and SEM.

### Assessment of ubiquitin, cyclins, CDKs, NF-kB by western blot assay

To test the inhibitory effect on proteasome activity, ubiquitin, cyclins, CDK, NF-kB and pNF-kB were assessed by western blot. Cells were seeded in 6-well plates and tested with the compounds (**5**–**6**) at the doses of 10 and 20 µM, based on the IC_50_ results. After 24 h, cells were collected and protein extracted for analysis. Protein extraction and western blot analysis were performed as previously described^12^.

Briefly, a total of 15 µg of proteins were loaded in each lane and specific antibodies were used to detect ubiquitin (Abcam, Cambridge, UK), cyclin A, cyclin B, cyclin D1, CDK1, CDK4/6, NF-kB, pNF-kB, PARP, and Caspase-3 (Cell Signaling, Danvers, MA, United States). Beta actin (Cell Signaling), was used on stripped blots as loading control. The images have been acquired using a dedicated software and densitometric data were expressed as integrated intensity and reported as means and SEM.

### Statistical analysis

All data are expressed as means ± SD Comparisons between different treatments were analysed by one-way or two-way ANOVA for non-parametric variables with Tukey’s post-test for intergroup comparisons. The possibility of error was set at *p* < .05 and it was considered statistically significant. All analyses were performed using Stata/IC 12.0 (StataCorp LP, College Station, TX). Graphs were drawn using GraphPad Prism (version 5.0 for Windows).

## Results and discussion

Compounds **1–6** were tested for their inhibitory properties on 20S immunoproteasome isolated from human spleen, using the appropriate fluorogenic substrate for each one of the proteolytic activities. First, compounds underwent a preliminary screening at 100 *µ*M using an equivalent volume of DMSO as a negative control, and MG-132, a reversible inhibitor of both proteasome and immunoproteasome, as positive control. Only amides **5** and **6**, characterised by the presence of a bromine-substituted naphthyridinone (i.e. **5**) or isoquinolinone (i.e. **6**) scaffold, respectively, showed an inhibition of one or two ChT-L activities > 70%, thus suggesting that the bromine could play a key role in establishing halogen bonds with residues of S3 pockets, in agreement to our previous findings on similar molecules.^7^

Thus amides **5** and **6** were characterised in detail, continuous assays were performed (progress curve method, at seven different concentrations, ranging from those that minimally inhibited to those that fully inhibited the immunoproteasome subunit) to determine the *K*_i_ values reported in Table 1. An analysis of the obtained *K*_i_ values clearly point out that compound **1** inhibited both the ChT-L activities (β5i and β1i) of i-20S to the same extent (*K*_i_ values of 4.39 µM and 4.90 µM, respectively). Differently amide **2**, was able to inhibit the sole β5i with a *K*_i_ value of 6.04 µM.

**Table 1.** Activity on immunoproteasome core-particles of amides **5**–**6**.

	*K*_i_ (µM) or % of inhibition at 100 µM		
Comp	*β*_1i_	*β*_2i_	*β*_5i_
**5**	36%	19%	6.04 ± 0.29
**6**	4.90 ± 0.04	24%	4.39 ± 0.18
**MG-132**	0.13 ± 0.01	0.0010 ± 0.00016	0.072 ± 0.014

**Figure 2.**
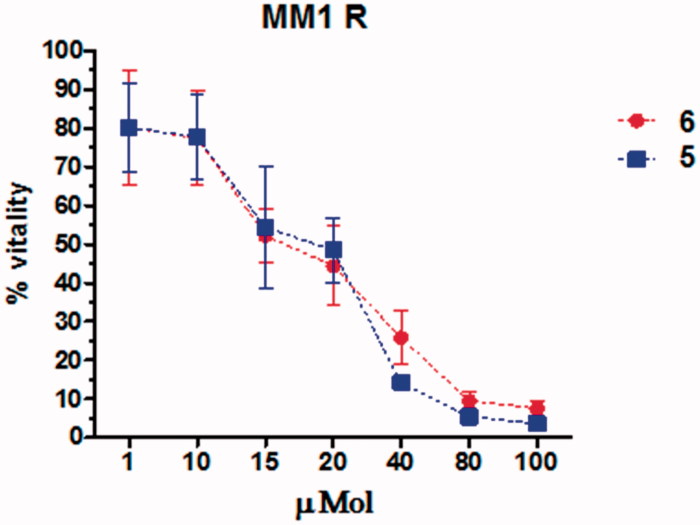
Effects of several concentrations of compounds **5** and **6** on vitality of MM.1R cells. Each point is obtained from the mean of 5 separate experiments.

To test the efficacy of compounds **5**–**6** in a cell system, we used dexamethasone-resistant human multiple myeloma cells (MM.1R), which are less responsive to chemotherapy, and are representative of patients in the later stages of the disease, as previously described^13^. All compounds were tested in duplicate at different doses (1, 10, 15, 20, 40, 80, 100 µM) and the cell survival assay was carried out at 24 h and repeated for 5 times. MM.1R cell survival was determined by the tetrazolium dye MTT assay and untreated cells were used as reference and assumed as the 100% of vital cells (Figure 2).

In this assay, compounds **5** and **6** were proven to be quite equipotent, with a slight improved activity for compound **6** (EC_50_ = 17.8 µM), with respect to amide **5** (EC_50_ = 21.2 µM).

**Figure 3.**
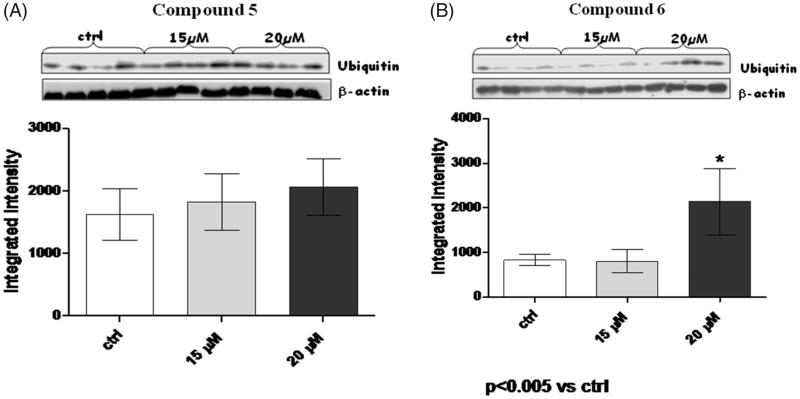
Effects of compounds **5**–**6,** in panels A and B respectively, on ubiquitin accumulation in MM.1R cells. The upper blots depicts ubiquitin (10 KDa) and the lower β-actin (45 KDa). Each bar is obtained from the mean of 3 separate experiments. *p < .005 versus ctrl.

To test the efficacy of these compounds in inhibiting ubiquitinated protein degradation, a western blot analysis of ubiquitin was performed (Figure 3). In fact, considering that proteasome inhibition leads the cells to accumulate ubiquitinated protein into the cytoplasm, we decided to assess the changes in levels of total ubiquitinated proteins indirectly, by detecting changes in total ubiquitin levels, by western blot analysis. In this case, a significant increase of ubiquitin (*p* < .005) was detected in the presence of compound **6** at 20 µM; on the contrary, no significant increase of ubiquitin accumulation was observed for compound **5** at none of the tested concentrations. It is worth noting that these changes were dose-dependent, this adding an additional level of confidence in our experimental approach.

Cyclin D1/CDK4 complex is crucial to promote G1/S transition^14^, so we decided to assess the levels of cyclin D1 and CDK4, as well as of other cell cycle progression markers such as Cyclin A, Cyclin B, and CDK1. As previously reported, proteasome inhibitors act as cytotoxic compounds by causing a reduction in both cyclins and CDKs levels, thus determining cell cycle arrest and apoptosis^15–16^.

**Figure 4.**
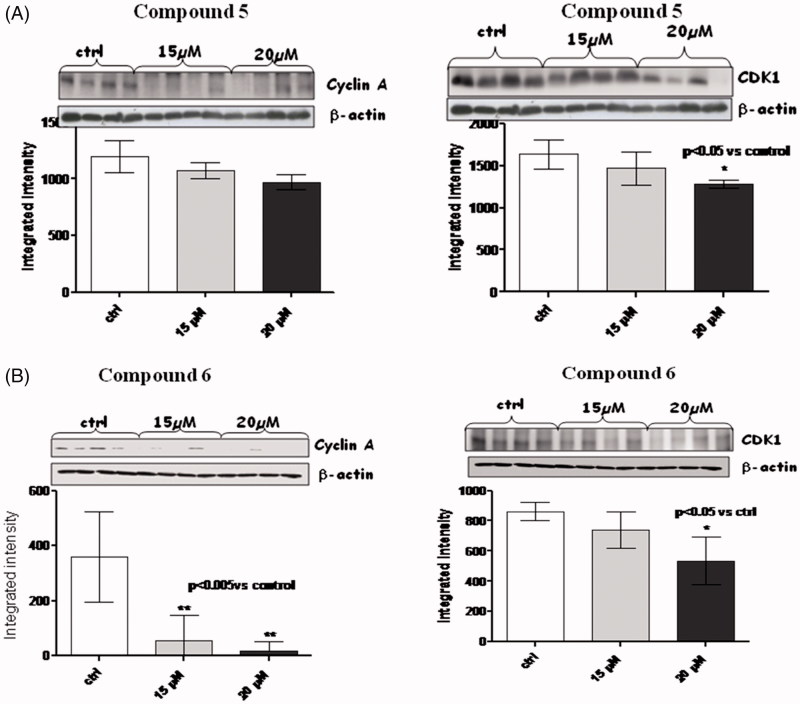
Figures depict western blots results relatively to cyclin A and CDK1, both normalized for β-actin (lower bands); panels A and B show results for compound **5** and **6**, respectively. Each bar is obtained from the mean of 3 separate experiments. *p < .005 versus ctrl; #*p* < .05 versus ctrl.

**Figure 5.**
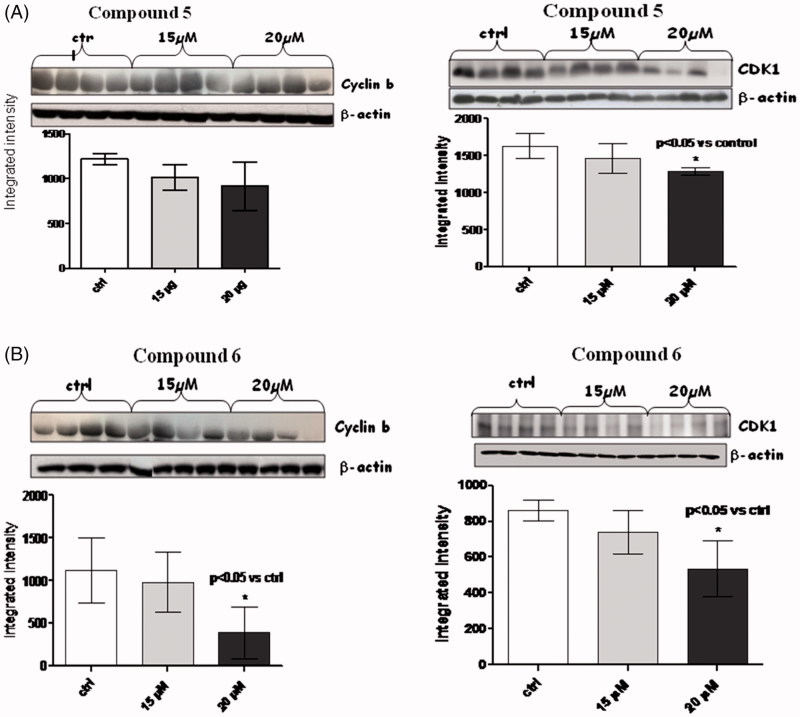
Figures depict western blots results relatively to cyclin B and CDK1, both normalized for β-actin (lower bands); panels A and B show results for compound **5** and **6**, respectively. Each bar is obtained from the mean of 3 separate experiments.

**Figure 6.**
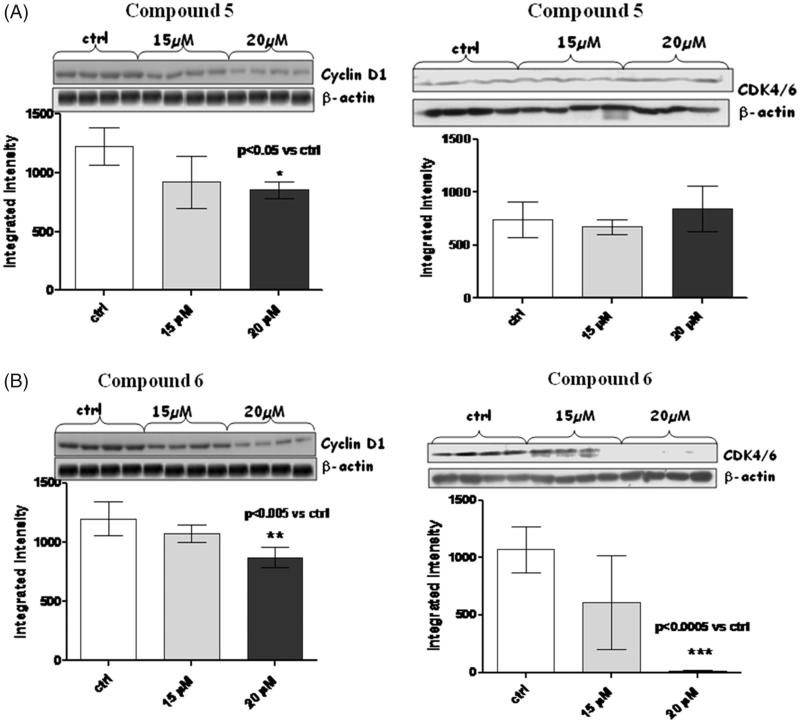
Figures depict western blots results relatively to cyclin D1 and CDK4/6, both normalized for β-actin (lower bands); panels A and B show results for compound **5** and **6**, respectively. Each bar is obtained from the mean of 3 separate experiments.

In the light of this, we decided to check whether our compounds (amides **5** and **6)** were able as well to act as negative regulators of cyclins and CDKs; we checked levels of cyclin D1, cyclin A, and cyclin B, as well as those of CDK1 and CDK4/6 (Figures 4–6).

When MM1R cells were exposed to 15 µM and 20 µM of amides **5** and **6** for a long-term incubation (24h), a very significant (*p* < .005) reduction in cyclin A (Figure 4) was observed only for compound **6** at both 15 and 20 µM. No trend of reduction was observed in this case for compound **5.**

A consistent reduction of the levels of cyclin B (Figure 5) and D1 (Figure 6) was also observed at 20 µM of compound **6,** while amide **5** was able to reduce only cyclin D1 at the same concentration.

In the case of compound **6**, the levels of cyclin A, B and D1 changed along with those of CDK-1 (*p* < .05) and CDK4/6 (*p* < .0005).

It is presumable to suppose that long-term incubation with compound **6**, leads to an inhibition of the cyclin A/CDK1, cyclin B/CDK1 and cyclin D1/CDK4/6 complexes, thus blocking cell cycle progression.

NF-kB transcription factors are primarily regulated by association with IkB protein. Thus, in most cells, NF-kB exists in the cytoplasm in an inactive complex bound to IkB. Most agents that activate NF-kB act through a common pathway based on phosphorylation-induced, proteasome-mediated degradation of IkB.

**Figure 7.**
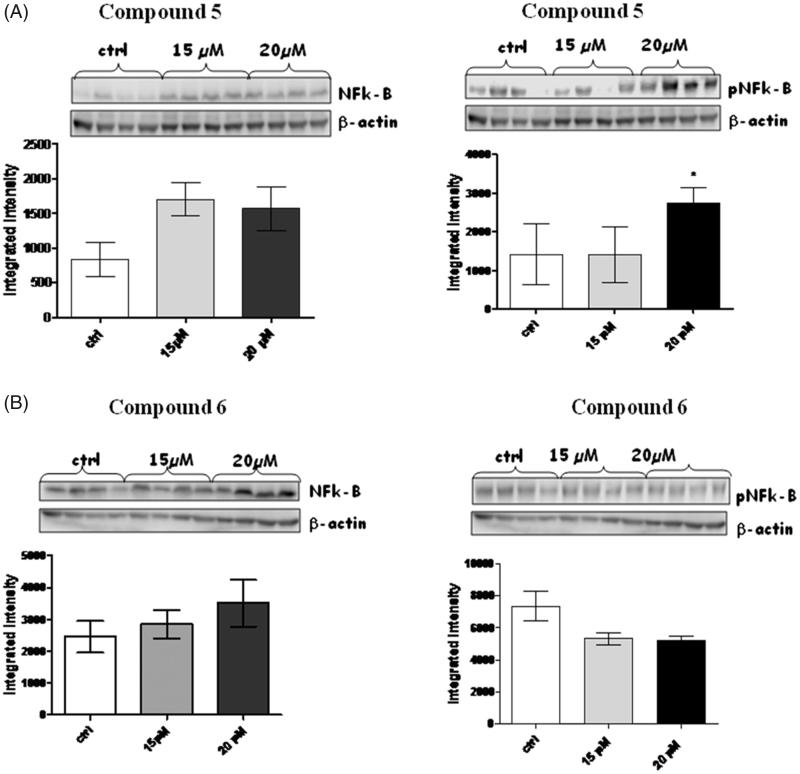
Figures depict western blots results relatively to NF-kB and phospho-NF-kB, both normalized for β-actin (lower bands); panels A and B show results for compound **5** and **6**, respectively. Each bar is obtained from the mean of 3 separate experiments.

With this in mind, western blot analysis were performed on both NF-kB and on its activated form pNF-kB (Figure 7), in this case, compound **6** induced a dose-dependent increase of NF-kB while, like expected, a trend of reduction was observed for pNF-kB.

On the contrary, a paradoxical increase of pNF-kB was observed with compound **5**; this effect has been already reported by several authors, and described in a plethora of cancer cell lines, as well as in primary cells from human cancer samples. There is still not a clear explanation of how proteasome inhibitors can activate, rather than inhibit NF-kB, but some authors hypothesised that this somehow surprising effect can be due by a differential NF-kB activation mechanism depending on type and/or dosage of the administered proteasome inhibitor^17–19^.

**Figure 8.**
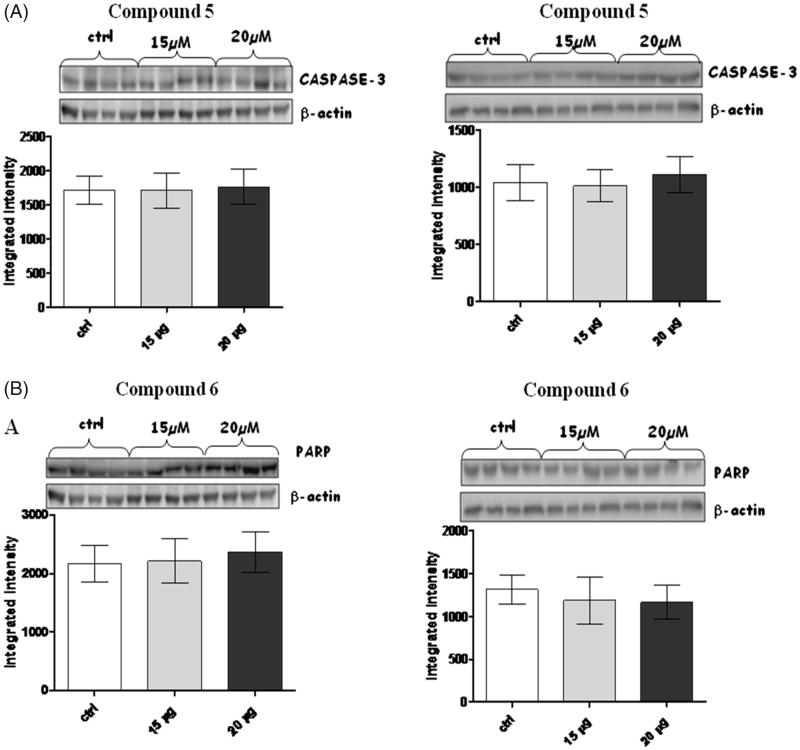
Figures depict western blots results relatively to PARP and Caspase-3, both normalized for β-actin (lower bands); panels A and B show results for compound **5** and **6**, respectively. Each bar is obtained from the mean of 3 separate experiments.

Apoptosis and necrosis are two cell death mechanisms that may be activated by PARP and Caspase-3. Therefore, PARP and Caspase-3 protein expression were studied to demonstrate that the efficacy of the compounds was not related to cell death processes^20–21^. No significant difference in PARP and Caspase-3 expression was observed using either compound 5 or 6 (Figure 8). These results demonstrated that the mechanism of action of these compounds is not related to apoptosis or necrosis.

## Conclusions

In conclusion, in our work, we identified two non-covalent immunoproteasome inhibitors, one of which (i.e. compound **6**) showed an ideal profile. Amide **6** was able to inhibit all the ChT-L activities of both proteasome and immunoproteasome, coupled with an EC_50_=17.8 µM against MM.1R cells, which are less responsive to chemotherapy and represent the late stage of the disease. Compound **6** was also able to inhibit ubiquitinated protein degradation, which is strictly related to proteasome inhibition. Lastly, it was proven to act on different phases of MM cell cycle reducing the levels of cyclin A/CDK1, cyclin B/CDK1 and cyclin D/CDK4/6 complexes, which turns in cell cycle arrest, nevertheless other mechanisms of cyclin and CDKs inhibition cannot be ruled out at this time. A trend of reduction of pNF-kB, accounts also this case for proteasome inhibition. Thus amide **6** can be certainly considered a promising lead compound for the discovery of novel noncovalent immunoproteasome inhibitors for the treatment of MM.

## References

[1] Micale N, Scarbaci K, Troiano V, et al. Peptide-based proteasome inhibitors in anticancer drug design. Med Res Rev 2014;34:1001–69. 24585725 10.1002/med.21312

[2] Ettari R, Previti S, Bitto A, et al. Immunoproteasome-selective inhibitors: a promising strategy to treat hematologic malignancies, autoimmune and inflammatory diseases. Curr Med Chem 2016;23:1217–38. 26965184 10.2174/0929867323666160318173706

[3] Ferrington DA, Gregerson DS Immunoproteasome: structure, function, and antigen presentation. Prog Mol Biol Transl Sci 2012;109:75–112. 22727420 10.1016/B978-0-12-397863-9.00003-1 PMC4405001

[4] Ettari R, Zappalà M, Grasso S, et al. Immunoproteasome-selective and non-selective inhibitors: a promising approach for the treatment of multiple myeloma. Pharmacol Ther 2018;182:176–92. 28911826 10.1016/j.pharmthera.2017.09.001

[5] Parlati F, Lee SJ, Aujay M, et al. Carfilzomib can induce tumor cell death through selective inhibition of the chymotrypsin-like activity of the proteasome. Blood 2009;114:3439–47. 19671918 10.1182/blood-2009-05-223677

[6] Ettari R, Bonaccorso C, Micale N, et al. Development of novel peptidomimetics containing a vinyl sulfone moiety as proteasome inhibitors. Chem Med Chem 2011;6:1228–37. 21506279 10.1002/cmdc.201100093

[7] Micale N, Ettari R, Lavecchia A, et al. Development of peptidomimetic boronates as proteasome inhibitors. Eur J Med Chem 2013;64:23–34. 23639651 10.1016/j.ejmech.2013.03.032

[8] Scarbaci K, Troiano V, Micale N, et al. Identification of a new series of amides as non-covalent proteasome inhibitors. Eur J Med Chem 2014;76:1–9. 24561716 10.1016/j.ejmech.2014.01.022

[9] Troiano V, Scarbaci K, Ettari R, et al. Optimization of peptidomimetic boronates bearing a P3 bicyclic scaffold as proteasome inhibitors. Eur J Med Chem 2014;83:1–14. 24946214 10.1016/j.ejmech.2014.06.017

[10] Scarbaci K, Troiano V, Ettari R, et al. Development of novel selective peptidomimetics, containing a boronic acid moiety, targeting the 20S proteasome as anticancer agents. Chem Med Chem 2014;9:1801–16. 24891205 10.1002/cmdc.201402075

[11] Di Giovanni C, Ettari R, Sarno SA, et al. Identification of noncovalent proteasome inhibitors with high selectivity for chymotrypsin-like activity by a multistep structure-based virtual screening. Eur J Med Chem 2016;121:578–91. 27318981 10.1016/j.ejmech.2016.05.049

[12] Pizzino G, Bitto A, Pallio G, et al. Blockade of the JNK signalling as a rational therapeutic approach to modulate the early and late steps of the inflammatory cascade in polymicrobial sepsis. Mediators Inflamm 2015;2015:1. 10.1155/2015/591572 PMC4385695 25873765

[13] Greenstein S, Krett NL, Kurosawa Y, et al. Characterization of the MM.1 human multiple myeloma (MM) cell lines: a model system to elucidate the characteristics, behavior, and signaling of steroid-sensitive and -resistant MM cells. Exp Hematol 2003;31:271–82. 12691914 10.1016/s0301-472x(03)00023-7

[14] Malara NM, Leotta A, Sidoti A, et al. Ageing, hormonal behaviour and cyclin D1 in ductal breast carcinomas. Breast 2006;15:81–9. 16473739 10.1016/j.breast.2004.12.008

[15] Huang X, Di Liberto M, Jayabalan D, et al. Prolonged early G(1) arrest by selective CDK4/CDK6 inhibition sensitizes myeloma cells to cytotoxic killing through cell cycle-coupled loss of IRF4. Blood 2012;120:1095–106. 22718837 10.1182/blood-2012-03-415984 PMC3412331

[16] Yang Z, Liu S, Zhu M, et al. PS341 inhibits hepatocellular and colorectal cancer cells through the FOXO3/CTNNB1 signaling pathway. Sci Rep 2016;6:22090. 26915315 10.1038/srep22090 PMC4768146

[17] Németh ZH, Wong HR, Odoms K, et al. Proteasome inhibitors induce inhibitory kappa B (I kappa B) kinase activation, I kappa B alpha degradation, and nuclear factor kappa B activation in HT-29 cells. Mol Pharmacol 2004;65:342–9. 14742676 10.1124/mol.65.2.342

[18] Dolcet X, Llobet D, Encinas M, et al. Proteasome inhibitors induce death but activate NF-kappaB on endometrial carcinoma cell lines and primary culture explants. J Biol Chem 2006;281:22118–30. 16735506 10.1074/jbc.M601350200

[19] Juvekar A, Manna S, Ramaswami S, et al. Bortezomib induces nuclear translocation of IκBα resulting in gene specific suppression of NFκB-dependent transcription and induction of apoptosis in CTCL. Mol Cancer Res 2011;9:183–94. 21224428 10.1158/1541-7786.MCR-10-0368 PMC3078042

[20] Tyagi A, Agarwal C, Harrison G, et al. Silibinin causes cell cycle arrest and apoptosis in human bladder transitional cell carcinoma cells by regulating CDKI-CDK-cyclin cascade, and caspase 3 and PARP cleavages. Carcinogenesis 2004;25:1711–20. 15117815 10.1093/carcin/bgh180

[21] Brauns SC, Dealtry G, Milne P, et al Caspase-3 activation and induction of PARP cleavage by cyclic dipeptide cyclo(Phe-Pro) in HT-29 cells. Anticancer Res 2005;25:4197–202. 16309216

